# Hepatic Compartment Syndrome Induced by Subcapsular Hematoma following Percutaneous Transhepatic Gallbladder Drainage: A Case Report

**DOI:** 10.70352/scrj.cr.26-0077

**Published:** 2026-06-03

**Authors:** Reina Miyazaki, Takanobu Hara, Hideki Ishimaru, Hajime Matsushima, Akihiko Soyama, Ayaka Kinoshita, Taiga Oka, Takashi Hamada, Hajime Imamura, Tomohiko Adachi, Susumu Eguchi

**Affiliations:** 1 Department of Surgery, Nagasaki University Graduate School of Biomedical Sciences, Nagasaki, Nagasaki, Japan; 2 Department of Radiology, Nagasaki University Hospital, Nagasaki, Nagasaki, Japan

**Keywords:** hepatic compartment syndrome, subcapsular liver hematoma, interventional radiology, percutaneous transhepatic biliary intervention

## Abstract

**INTRODUCTION:**

Hepatic compartment syndrome (HCS) is a rare but potentially fatal condition caused by acute elevation of intrahepatic pressure, most often secondary to a subcapsular hematoma. Iatrogenic cases, particularly after percutaneous hepatobiliary interventions, have been only sporadically reported. We describe HCS following percutaneous transhepatic gallbladder drainage (PTGBD) complicated by a subcapsular hepatic hematoma.

**CASE PRESENTATION:**

A 49-year-old man underwent PTGBD for acute cholecystitis at the referring hospital. The catheter dislodged, and repeated attempts at re-placement over a guidewire failed, leaving the tip within the hepatic subcapsular space. The following day, he developed worsening abdominal pain, and CT revealed a massive right hepatic subcapsular hematoma. He was urgently transferred to our hospital. Contrast-enhanced CT demonstrated active extravasation, prompting emergency interventional radiology. Hepatic arteriography showed hepatofugal flow in the portal venous system, confirming HCS caused by intraparenchymal hypertension due to subcapsular compression. Decompression was achieved by placing a drain into the hematoma via the original PTGBD tract, followed by selective hepatic arterial embolization. Peak serum aspartate aminotransferase and alanine aminotransferase levels reached 9873 and 7385 U/L, respectively, before gradually declining. The hematoma caused compression of the inferior vena cava, intra-abdominal hypertension, and secondary acute kidney injury with pulmonary edema, requiring temporary renal replacement therapy. The hematoma progressively regressed with restored hepatic perfusion. Renal replacement therapy was discontinued prior to transfer, the drain was removed on day 24, and the patient was transferred for rehabilitation on day 34.

**CONCLUSIONS:**

Early recognition of HCS and prompt decompression are essential to prevent irreversible hepatic and multiorgan injury.

## Abbreviations

ACS
: abdominal compartment syndrome
CHDF
: continuous hemodiafiltration
HCS
: hepatic compartment syndrome
PTGBD
: percutaneous transhepatic gallbladder drainage
PT-INR
: prothrombin time–international normalized ratio
TAE
: transarterial embolization

## INTRODUCTION

HCS is a rare but potentially fatal condition characterized by a rapid increase in intrahepatic pressure, often resulting from a subcapsular or intraparenchymal hematoma.^1–6)^ Elevated intrahepatic pressure can compromise hepatic venous outflow and portal perfusion, leading to acute liver failure and, in severe cases, multiorgan dysfunction.^1,3,4)^

Iatrogenic HCS has been reported after hepatobiliary interventions, including liver biopsy and surgeries in the right upper abdominal region.^1–3,6–8)^ In contrast, HCS associated with PTGBD is exceedingly uncommon, with very limited documentation.^4)^ We herein report a case of HCS caused by a massive subcapsular hematoma following PTGBD, which was successfully managed with percutaneous decompression and TAE.

## CASE PRESENTATION

A 49-year-old man with a history of brainstem hemorrhage and hypertension, but no history of liver disease, presented with acute right-sided flank pain. He was not receiving anticoagulant or antiplatelet therapy. Twelve days before admission to our hospital, he underwent endoscopic biliary stent placement for choledocholithiasis at the referring hospital. He subsequently developed acute cholecystitis and underwent PTGBD. At the time of PTGBD placement, coagulation parameters were within normal limits (PT-NIR, 1.06; platelet count, 328 × 10^3^/μL), although activated partial thromboplastin time was not measured. Five days later, a decrease in drainage from the PTGBD catheter prompted an intraductal cholangiography, which confirmed the catheter tip had migrated into the subhepatic space. Image-guided catheter repositioning using a guidewire was attempted, but the guidewire followed an abnormal path, raising suspicion of displacement into the abdominal cavity. Subsequently, the catheter was clamped, and a CT was performed. This confirmed the tip was located subcapsularly, but no obvious active leakage was detected, leading to a decision for observation. The following day, abdominal pain worsened. A CT scan performed at the referral hospital revealed a massive subcapsular hepatic hematoma, necessitating emergency transport. This bleeding was considered to be caused by the repeated and prolonged guidewire manipulation during the repositioning attempt.

On arrival, his height and weight were 165 cm and 64.5 kg, respectively. Vital signs were SpO_2_ 96% on room air, respiratory rate 20/min, heart rate 72/min, blood pressure 122/92 mmHg, and body temperature 38.0°C. His Glasgow Coma Scale score was E4V5M6. Physical examination revealed icteric sclera, generalized jaundice, and marked tenderness over the right flank.

Initial laboratory tests showed significant liver dysfunction with aspartate aminotransferase (AST) 1301 U/L, alanine aminotransferase (ALT) 834 U/L, total bilirubin 7.2 mg/dL (direct bilirubin 5.5 mg/dL), and alkaline phosphatase 171 U/L. Inflammatory markers were elevated (white blood cell 14100/µL; C-reactive protein 2.40 mg/dL). He was anemic (hemoglobin 10.1 g/dL) and had coagulopathy (PT-INR 2.02). Serum creatinine was 1.17 mg/dL, indicating early renal impairment.

**Fig. 1:**
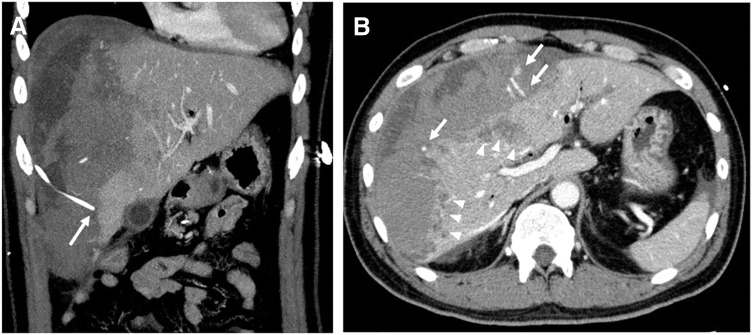
Contrast-enhanced CT of the abdomen. (**A**) The PTGBD catheter tip (arrow) is displaced into the subcapsular space of the right hepatic lobe, with a large subcapsular hematoma. (**B**) The hematoma causes marked mass effect on the hepatic parenchyma with collapse of the inferior vena cava and multiple adjacent non-enhancing hepatic areas (arrowheads) suggestive of ischemia. Several foci of active contrast extravasation (arrows) are also evident along the hepatic surface. PTGBD, percutaneous transhepatic gallbladder drainage

Abdominal contrast-enhanced CT demonstrated that the PTGBD tube tip had migrated into the subcapsular space of the right hepatic lobe, forming a large subcapsular hematoma (**Fig. 1A**). The hematoma compressed the hepatic parenchyma, causing collapse of the inferior vena cava, and multiple non-enhancing areas were noted in the underlying liver, suggesting ischemia. Several foci of active extravasation were also identified (**Fig. 1B**). Given the presence of a large hematoma with ongoing bleeding, urgent hemostasis was required. Because he remained hemodynamically stable, TAE was selected as the initial intervention.

**Fig. 2:**
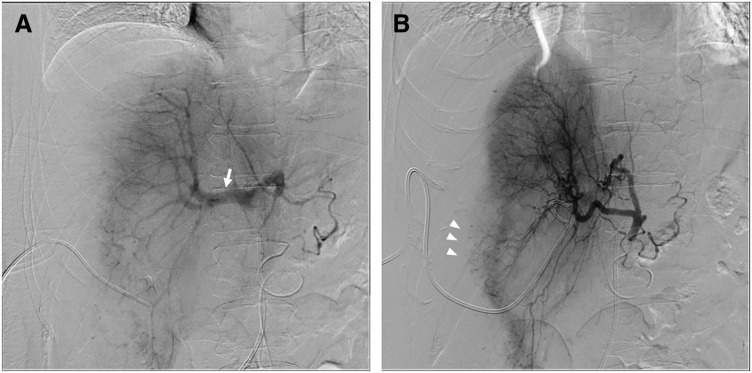
Hepatic angiography. (**A**) Hepatic arteriography shows hepatofugal flow—retrograde opacification of the portal vein during the arterial phase (arrow)—consistent with HCS. (**B**) Under compression from the tense subcapsular hematoma, active extravasation was initially inconspicuous; after percutaneous decompression via hematoma drainage, multiple foci of extravasation along the hepatic surface became clearly visible (arrowheads). HCS, hepatic compartment syndrome

Hepatic artery angiography demonstrated hepatofugal flow into the portal vein, a characteristic finding of HCS (**Fig. 2A**). This finding was observed during right hepatic artery angiography. In contrast, hepatopetal flow was preserved in the left portal vein. This finding suggests localized impairment of portal inflow due to marked compression of the right hepatic parenchyma by the subcapsular hematoma. To achieve decompression prior to TAE, the pre-existing PTGBD catheter was exchanged for a PTCD kit (drainage tube, 7 Fr, 350 mm, Create Medic, Yokohama, Kanagawa, Japan), which initially drained approximately 50 mL of blood. Decompression rendered previously occult extravasation more apparent (**Fig. 2B**), and selective right hepatic arterial embolization was performed, successfully eliminating the extravasation.

**Fig. 3:**
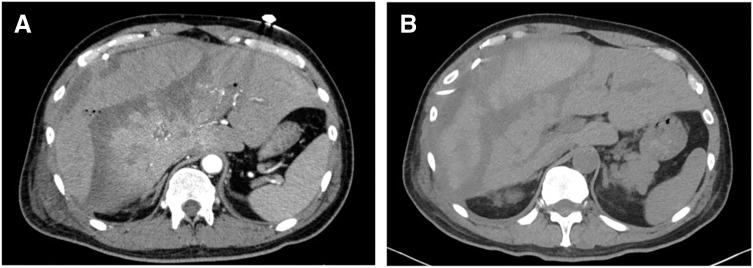
Follow-up contrast-enhanced CT. (**A**) Contrast-enhanced CT performed 3 days after TAE shows the disappearance of contrast extravasation, indicating successful hemostasis. (**B**) Follow-up CT on day 21 demonstrates regression of the subcapsular hematoma and hepatic infarction. TAE, transarterial embolization

**Fig. 4:**
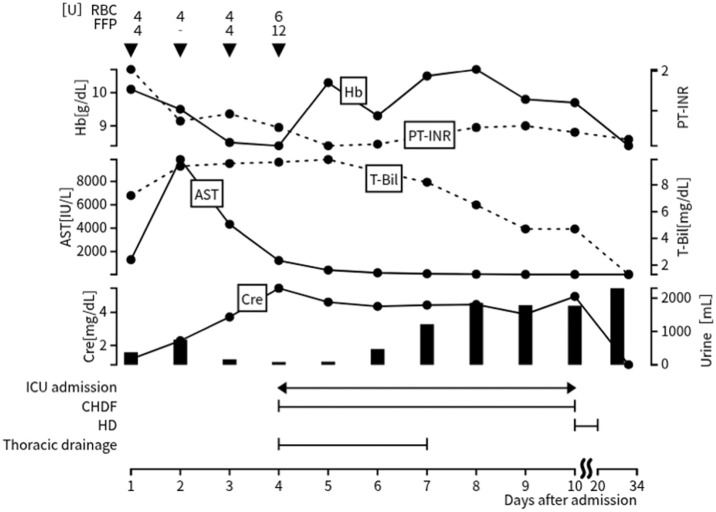
Clinical course. Trends in Hb, PT-INR, AST, T-Bil, and serum Cre after admission. Bars indicate daily urine output. Periods of CHDF and intermittent HD are shown. Owing to progression of acute kidney injury and pulmonary edema, the patient was admitted to the ICU on day 4, when thoracic drainage and CHDF were initiated; CHDF was subsequently switched to HD between days 10 and 20. The patient was transferred to a rehabilitation hospital on day 34. AST, aspartate aminotransferase; CHDF, continuous hemodiafiltration; Cre, creatinine; FFP, fresh frozen plasma; Hb, hemoglobin; HD, hemodialysis; PT-INR, prothrombin time–international normalized ratio; RBC, red blood cell; T-Bil, total bilirubin

On the following day, serum AST peaked at 9873 U/L and ALT peaked at 7385 U/L, indicating severe acute liver injury, but gradually improved thereafter. Follow-up contrast-enhanced CT performed 3 days after TAE showed the disappearance of contrast extravasation (**Fig. 3A**). Daily transfusions were administered for anemia and coagulopathy. He developed anuria and acute kidney injury due to renal hypoperfusion and hypovolemia associated with HCS. Fluid resuscitation induced pulmonary edema, necessitating ICU admission and initiation of CHDF. His respiratory status improved with thoracic drainage and nasal high-flow therapy. CHDF was discontinued on day 20 of hospitalization. Follow-up CT on day 21 demonstrated a decrease in hematoma volume and hepatic infarction (**Fig. 3B**). The subcapsular drain was removed on day 24, and the patient was transferred to a rehabilitation facility on day 34, fully weaned from renal replacement therapy (**Fig. 4**).

## DISCUSSION

HCS is an exceedingly rare clinical entity characterized by a rapid and pathological increase in intrahepatic parenchymal pressure, which impairs intrahepatic perfusion and precipitates acute liver dysfunction. The concept was first introduced by Pearl and Trunkey in 1999 in a case report describing HCS as a complication of hepatic trauma.^9)^ Although most reported cases have followed blunt or penetrating hepatic trauma, HCS has also been associated with iatrogenic interventions such as percutaneous hepatobiliary procedures and right upper abdominal surgery, including liver resection^1–20)^ (**Table 1**). Characteristic findings of HCS include a sudden and marked elevation of serum aminotransferase levels, non-enhancing areas of the hepatic parenchyma on contrast-enhanced CT indicating ischemia or necrosis, and hepatofugal flow—retrograde opacification of the portal vein during the arterial phase on hepatic arteriography—reflecting severely elevated intrahepatic pressure and compromised microcirculation.^10)^

**Table 1:** Reported cases of HCS

Case	Author/year	Age/sex	Cause	Time from injury to HCS	HFF	Treatment	Hemodynamic instability before treatment	Complications	Peak AST/ALT (IU/L)	Reported outcome
1	Pearl and Trunkey/1999^9)^	15/F	Trauma	8 h	+	Surgery (subcapsular hematoma evacuation)	No	ND	718/913	Discharged, day 5
2	Kon et al./2002^11)^	23/M	Liver cyst developed after trauma	6 months	–	Surgery (fenestration and debridement)	No	None	ND	Alive without symptoms, 1 year
3	Hayakawa et al./2005^10)^	40/F	Trauma	6 h	+	TAE	No	None	2971/1930	Alive without symptoms, 6 months
4		73/M	Trauma	9 h	+	TAE	No	None	2217/1876	Alive without symptoms, 6 months
5	Tohma et al./2007^12)^	36/F	Trauma	12 h	+	Surgery (subcapsular hematoma evacuation)	No	None	5663/3952	Discharged, day 38
6		14/F	Trauma	12 h	+	Percutaneous drainage	No	None	2391/3108	Discharged, day 32
7	Sueyoshi et al./2008^13)^	60/F	Liver injury after CPR	2 days	–	TAE	No	None	10029/6885	Discharged, day 73
8	Nissen et al./2010^7)^	28/F	Percutaneous liver biopsy after LDLT	20 h	–	Re-LT	Yes	Acute renal failure	>6000/>8000	Discharged, day 13
9	Ye and Miao/2014^14)^	35/F	Liver surgery for trauma	16 days	ND	Percutaneous drainage	No	None	—/878	Discharged, day 47
10	Fujiyoshi et al./2015^15)^	40/F	Liver injury after CPR	24 h	–	Conservative treatment	Yes	None	1905/1640	Discharged, day 21
11	Marcaire et al./2016^4)^	64/M	PTGBD	15 days	ND	TAE followed by percutaneous drainage	Yes	Multiple organ failure	13897/3350	Died (multiple organ failure), 2 months
12	Sato et al./2016^16)^	19/M	Biloma developed after trauma	9 days	ND	TAE followed by percutaneous drainage	No	Adhesive intestinal obstruction	1213/1060	Discharged, day 41
13	Ando et al./2017^17)^	32/F	Spontaneous	ND	+	TAE followed by percutaneous drainage	No	None	819/957	Alive without symptoms, 9 months
14	Lee et al./2019^2)^	25/M	Laparoscopic adrenalectomy	2 days	ND	Percutaneous drainage followed by TAE	No	None	2953/2153	Discharged, day 7
15	Masui et al./2019^18)^	36/F	Trauma	40 h	+	Surgery (subcapsular hematoma evacuation)	No	None	5663/3952	Discharged, day 38
16		14/F	Trauma	28 h	+	Percutaneous drainage	ND	ND	2391/3108	ND
17		40/M	Trauma	ND	+	Percutaneous drainage	ND	ND	21000/7900	ND
18		20/M	Trauma	9 h	ND	Surgery (subcapsular hematoma evacuation)	ND	ND	4654/4853	ND
19		15/M	Trauma	65 h	+	TAE followed by percutaneous drainage	No	None	928/977	Discharged, day 19
20	Takaoka et al./2019^19)^	19/F	Trauma	32 h	+	TAE	No	None	4237/1773	Alive without symptoms, 1 year
21		52/M	Trauma	65 h	+	TAE	No	None	10682/6989	Discharged, not specified
22	Foran et al./2020^8)^	71/M	Diaphragm plication	20 h	+	Surgery (removal of sutures from the diaphragm)	Yes	None	5581/3347	Discharged, day 7
23	Lacroix et al./2022^3)^	38/F	Laparoscopic nephrectomy	Few hours	+	Surgery (subcapsular hematoma evacuation)	Yes	None	ND	Discharged, not specified
24	Chang et al./2023^6)^	59/F	Diaphragm plication	2 days	–	Conservative	No	None	4099/2303	Recovered at day 6
25	Fujita et al./2023^5)^	12/M	Trauma	2 days	ND	Surgery (subcapsular hematoma evacuation + packing) followed by TAE	Yes	None	ND	ND (9 days of ICU stay)
26	Nassar et al./2024^1)^	54/M	Trauma	4 days	ND	Surgery (subcapsular hematoma evacuation + packing)	Yes	Acute renal failure, pneumonia	9658/7215	Alive, 16 days
27		56/M	DDLT	2 days	ND	Surgery (subcapsular hematoma evacuation)	Yes	Renal failure, respiratory insufficiency, histoplasmosis	285 ULL/66 ULN	Died (histoplasmosis), 4 months
28		59/M	Laparoscopic cholecystectomy	5 days	ND	Surgery (subcapsular hematoma evacuation + packing)	No	Pneumonia	10 ULN/6 ULN	Discharged, day 20
29	Okura/2023^20)^	21/M	Trauma	12 h	+	TAE	No	None	2190/3411	Discharged, day 20

ALT, alanine aminotransferase; AST, aspartate aminotransferase; CPR, cardiopulmonary resuscitation; DDLT, deceased donor liver transplantation; F, female; HCS, hepatic compartment syndrome; HFF, hepatofugal flow; LDLT, living donor liver transplantation; M, male; ND, not described; PTGBD, percutaneous transhepatic gallbladder drainage; Re-LT, re-liver transplantation; TAE, trans arterial embolization; ULL, upper level of normal; ULN, upper limit of normal

In the present case, HCS developed secondary to an iatrogenic subcapsular hepatic hematoma following PTGBD. Although the exact rationale for guidewire-assisted reinsertion at the referring hospital remains unknown, concern about bile leakage or bleeding after early catheter removal may have influenced the decision to avoid immediate tube withdrawal. However, this case illustrates that repeated and forceful guidewire manipulation in the setting of early PTGBD dislodgement likely injured small intrahepatic arterial branches, resulting in active bleeding and subcapsular hematoma formation. The rapidly expanding hematoma caused a marked elevation in intrahepatic pressure, which compromised hepatic perfusion and triggered the cascade characteristic of HCS. Although TAE successfully achieved hemostasis, the hematoma persisted as a large space-occupying lesion. The extensive subcapsular hematoma along the lateral aspect of the right hepatic lobe exerted a significant mass effect on the inferior vena cava, resulting in venous outflow obstruction and reduced venous return. The ensuing venous congestion and relative hypovolemia precipitated acute kidney injury through decreased renal perfusion. Massive transfusions and aggressive fluid resuscitation contributed to pulmonary edema and respiratory failure, necessitating renal replacement therapy for fluid removal, together with respiratory support using nasal high-flow therapy. Although ACS was not strongly suspected in the present case, intra-bladder pressure measurement might have been useful for differentiating isolated HCS from concomitant ACS in the setting of intra-abdominal mass effect and multiorgan dysfunction. After exchange of the PTGBD catheter for a hematoma drain, the initial drainage volume was approximately 240 mL and subsequently decreased, whereas bloody ascites increased and required additional abdominal drainage. These findings illustrate how localized hepatic compartmental hypertension can propagate into multiorgan dysfunction through complex hemodynamic interactions.

**Table 1** summarizes the literature review. Between 1999 and 2024, 29 cases were described in the literature. In the collated series, trauma-related events accounted for the majority of reports, with right-lobe subcapsular hematoma predominating. The interval from inciting event to HCS spans hours to over 2 weeks, underscoring that HCS can evolve both acutely and subacutely. Peak aminotransferases frequently rise into the thousands (occasionally >10000 U/L), reflecting abrupt hepatocellular injury. Management strategies cluster into 3 approaches: (i) surgical evacuation with or without gauze packing, (ii) TAE, and (iii) percutaneous decompression—often used in combination-related cases. Outcomes are generally favorable when rapid decompression and hemostasis are achieved, although complications (e.g., acute kidney injury) and ICU stays are common in severe presentations. Nontraumatic and iatrogenic triggers are increasingly recognized. Reports include posttransplant, laparoscopic nephrectomy, laparoscopic adrenalectomy, diaphragm plication, and laparoscopic cholecystectomy, consistent with the notion that right upper abdominal surgery can initiate HCS via subcapsular hemorrhage and abrupt intraparenchymal pressure rise. By contrast, HCS following PTGBD is exceedingly uncommon, with very limited documentation.^4)^ In the available reports, combined percutaneous drainage and TAE was attempted, yet multiorgan failure occurred.^4)^ By contrast, our patient underwent prompt decompression via the PTGBD tract followed by selective TAE, with recovery of hepatic perfusion and successful weaning from renal replacement therapy, highlighting that early, staged, minimally invasive decompression plus targeted hemostasis can avert progression to irreversible liver injury and multiorgan failure even when the precipitating factor is iatrogenic.

In our case, a key management question was whether surgical intervention should have been pursued. Because active extravasation was present on contrast-enhanced CT and the patient remained hemodynamically stable, we selected staged nonoperative management with percutaneous decompression followed by targeted TAE as the initial strategy. This approach achieved timely hemostasis, reduced intrahepatic pressure, and was followed by improvement in liver injury markers after their peak on the next day. However, this should not be interpreted as indicating that surgery was unnecessary. The residual hematoma continued to compress the inferior vena cava, and the patient subsequently required intensive care for acute kidney injury and respiratory failure. Surgical hematoma evacuation was therefore considered throughout the clinical course and would likely have been performed if liver dysfunction had worsened further or if respiratory or circulatory failure had become refractory. In retrospect, delayed hematoma evacuation after initial hemostasis and stabilization might also have been a reasonable alternative strategy. At the same time, immediate surgery in this setting would likely have been challenging because identifying a discrete bleeding point within a large hematoma may have been difficult, with a possibility of requiring perihepatic packing or even open abdominal management. Thus, while percutaneous decompression and TAE may be prioritized initially in hemodynamically stable patients, surgical evacuation should not be delayed when improvement in liver function or overall clinical status is not promptly achieved.

In summary, in a stable patient with HCS physiology and active bleeding, decompression is performed first to unmask the culprit lesion, immediately followed by selective TAE. This constitutes a rational and effective strategy, obviating the need for surgical decompression while enabling recovery of hepatic perfusion and successful weaning from renal replacement therapy.

## CONCLUSIONS

Early diagnosis of HCS and appropriate selection of subsequent treatment are crucial. HCS should be considered early when clinical signs such as severe right-sided pain, abrupt transaminase surge, and imaging consistent with a large subcapsular hematoma are present. In hemodynamically stable patients, a staged nonoperative management—percutaneous decompression to relieve intrahepatic pressure, followed by selective TAE for hemostasis—can be decisive and life-saving. Prompt, physiology-guided intervention and coordinated critical care are essential to prevent irreversible liver injury and multiorgan failure.

## DECLARATIONS

### Availability of data and materials

The datasets supporting the conclusions of this article are included within the article.
